# Investigating the reliability and validity of subacromial space measurements using ultrasound and MRI

**DOI:** 10.1186/s13018-023-04482-1

**Published:** 2023-12-22

**Authors:** Stephen M. Boulanger, Alexandra Mahna, Talia Alenabi, Anthony A. Gatti, Oriana Culig, Loriann M. Hynes, Jaclyn N. Chopp-Hurley

**Affiliations:** 1 School of Kinesiology and Health Science, York University, 4700 Keele Street, Toronto, ON M3J 1P3 Canada; 2 Department of Kinesiology, University of Waterloo, Waterloo, ON Canada; 3 Department of Radiology, Stanford University, Stanford, CA USA; 4 NeuralSeg Ltd., Hamilton, ON Canada

**Keywords:** Shoulder, Reliability, Magnetic resonance imaging, Ultrasound

## Abstract

**Background:**

While ultrasound (US) measures of the subacromial space (SAS) have demonstrated excellent reliability, measurements are typically captured by experts with extensive ultrasound experience. Further, the agreement between US measured SAS width and other imaging modalities has not been explored. This research evaluated the agreement between SAS measures captured by novice and expert raters and between US and magnetic resonance imaging (MRI). This study also evaluated the effect of US transducer tilt on measured SAS.

**Methods:**

Nine men and nine women participated in this study. US images were captured by a novice and expert with the participant in both seated and supine positions. An inclinometer was fixed to the US probe to measure transducer tilt. SAS width was measured in real time from freeze framed images. MRI images were captured, and the humerus and acromion manually segmented. The SAS width was measured using a custom algorithm.

**Results:**

Intraclass correlation coefficients (ICCs) between novice and expert raters were 0.74 and 0.63 for seated and supine positions, respectively. Intra-rater agreement was high for both novice (ICC = 0.83–0.84) and expert (ICC ≥ 0.94) raters. Agreement between US and MRI was poor (ICC = 0.21–0.49) but linearly related.

**Conclusions:**

Moderate agreement between novice and expert raters was demonstrated, while the agreement between US and MRI was poor. High intra-rater reliability within each rater suggests that US measures of the SAS may be completed by a novice with introductory training.

## Background

The subacromial space (SAS) is comprised of the humeral head inferiorly, and the anteroinferior surface of the anterior third of the acromion, the coracoacromial ligament and acromioclavicular joint superiorly [1]. Reduction of the SAS can lead to subacromial impingement syndrome (SAIS), whereby the tissues occupying the space, notably the supraspinatus tendon, become compressed and subsequently damaged [2]. SAIS is one of the most common shoulder disorders, accounting for 48% of all clinical diagnoses [3]. Narrowing of the SAS can result from musculoskeletal exposures that modify healthy joint kinematics [2, 4–6] or innate morphological parameters [7–9]. Both mechanisms pose risk of SAIS and/or rotator cuff tears and subsequent pain, functional limitations, and further tissue injury [10–12]. Thus, accurate measurement of the SAS width is beneficial for risk identification and prevention.

A variety of imaging-based methods have been used to quantify the SAS. Radiography has been used to capture the acromiohumeral interval (minimum SAS width), demonstrating high reliability [8, 13–16]. However, changes in arm position and X-ray beam orientation have been shown to overestimate SAS measurements [14, 17]. While radiography captures a single static image, biplane fluoroscopy enables investigators to view the structures dynamically across simultaneous images [18]. This method is particularly advantageous as it offers the same benefits as conventional radiography, while allowing for three-dimensional measurement of the SAS in both static and dynamic states [18, 19]. Researchers investigating the SAS have also used computed tomography (CT) scans, which yields greater precision compared to conventional radiography [20, 21]. Bey and colleagues [19, 22–24] developed a model-based motion tracking technique that uses CT scans alongside biplane radiographs to track in vivo glenohumeral kinematics with a high degree of accuracy compared to dynamic radiostereometric analysis (RMS errors < 0.385 mm for the scapula and < 0.374 mm for the humerus) [24]. This method has been subsequently adopted by other research groups and deemed the gold standard for quantifying the SAS [25]. However, while deemed noninvasive, these techniques still expose participants to radiation, albeit the effective dosage is reportedly low [25]. Magnetic resonance imaging (MRI) has been regarded as the most appropriate imaging technique for complex anatomical structures [26–28]. Research using MRI to capture the SAS width reports high precision, reliability, and accuracy of the anatomical models reconstructed from MRI images [27, 29]. While these methods have been deemed effective for measuring the SAS, they are not readily accessible and can be expensive. Ultrasound (US) is another imaging modality that has been used to measure the SAS. US is efficient, noninvasive, relatively easy to administer, and inexpensive. However, US may overestimate the minimum SAS width, as the lateral positioning of the probe may capture the distance between the acromion and lateral humerus rather than the minimum distance between the coracoacromial arch and the superior-most point on the humeral head.

Several studies have investigated the inter- and intra-rater reliability of SAS measures using US. Among studies where the agreement in SAS measures between experienced clinicians was quantified, excellent intra- (ICC: ≥ 0.92) and inter- (ICC: ≥ 0.88) rater reliability was reported [30–32]. In these studies, participants included asymptomatic individuals, and patients with SAIS and/or rotator cuff tendinopathy. Further, excellent within-day (ICC ≥ 0.98) and day-to-day (ICC ≥ 0.96) intra-rater reliability has been reported for expert raters [33]. Other studies have compared the agreement of SAS measurements captured by both novice and expert raters. Novice raters included physical therapists, physiotherapy students, and orthopedic residents who received practice and/or training prior to commencing the study [34–37]. These studies revealed that the novice raters demonstrated good to excellent inter-rater reliability among one another (ICC: 0.79) [35] and compared to an expert (0.70–0.77) [34, 36, 37]. Intra-rater reliability for the novice rater was reported to be excellent (ICC ≥ 0.84) [35, 37]. SAS measurement agreement between expert clinicians and non-clinician novice raters, as well as the agreement between their US measures and that quantified from MRI, has not been studied.

In an effort to improve reliability of US-based measures, researchers have implemented various tools and methodologies. Bulbrook et al. [38] introduced a 3D-printed novel transducer attachment to improve the reliability of capturing muscle architecture parameters in the thigh. In the shoulder, an inclinometer has been attached to the US probe in effort to assist in maintaining a consistent probe angle between repeated images [39]. However, researchers have not studied whether SAS measures captured by two raters are in better agreement when the same transducer angle is adopted. Although measurements performed by expert raters have previously demonstrated excellent reliability, minimal research exists that examines the validity of SAS compared to other imaging modalities and the sensitivity to transducer tilt variation. It is also important to determine whether a novice non-clinician rater with minimal US training can reliably and accurately measure the SAS. Confirmed reliability of a novice researcher, as compared to an expert clinician, and validity, as compared to MRI, would enable more feasible and accessible laboratory-based assessments of SAIS risk.

The primary purposes of this study were to: (1) evaluate the agreement of SAS measures captured using US between a novice with introductory training and an expert and (2) evaluate the agreement between US and MRI measures of the SAS. We hypothesized that the SAS measurements of novice and expert raters would have moderate to excellent agreement. We also expected the US measures to overestimate the minimum SAS captured from MRI due to the imaging capabilities of US. The secondary purpose of this research was to determine whether inter-rater reliability was improved when the transducer tilt angle was consistent between raters. We hypothesized that inter-rater reliability would be improved when a consistent transducer tilt angle was adopted.

## Methods

### Participants

Eighteen right-hand dominant, healthy young adults (9 M, 9W; 23.6 (2.6) years; 24.9 (3.4) kg/m^2^) free from self-reported shoulder pain or injury in their right shoulder were recruited from the university population. Participants were excluded if they had positive signs for any of three clinical impingement tests (Hawkins-Kennedy, Painful Arc Sign and/or Infraspinatus Muscle Strength Tests) [40]. In addition, participants were excluded if they had a Disabilities of the Arm, Shoulder, and Hand (DASH) score > 25 [41]. Participants were also excluded if it was deemed unsafe for them to enter the MRI room; this included having any metal within their body (i.e., implanted clips or devices) or were pregnant. This study received ethical approval through the institutional research ethics board (York University Office of Research Ethics #e2019-387).

### MRI: acquisition and analysis

**Fig. 1:**
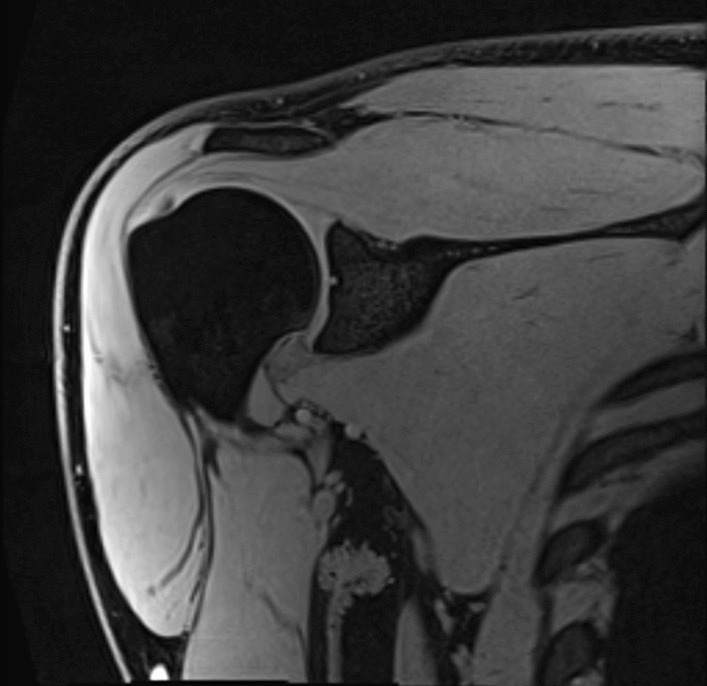
MRI image of the right shoulder using a T1 VIBE sequence in the coronal plane

**Fig. 2:**
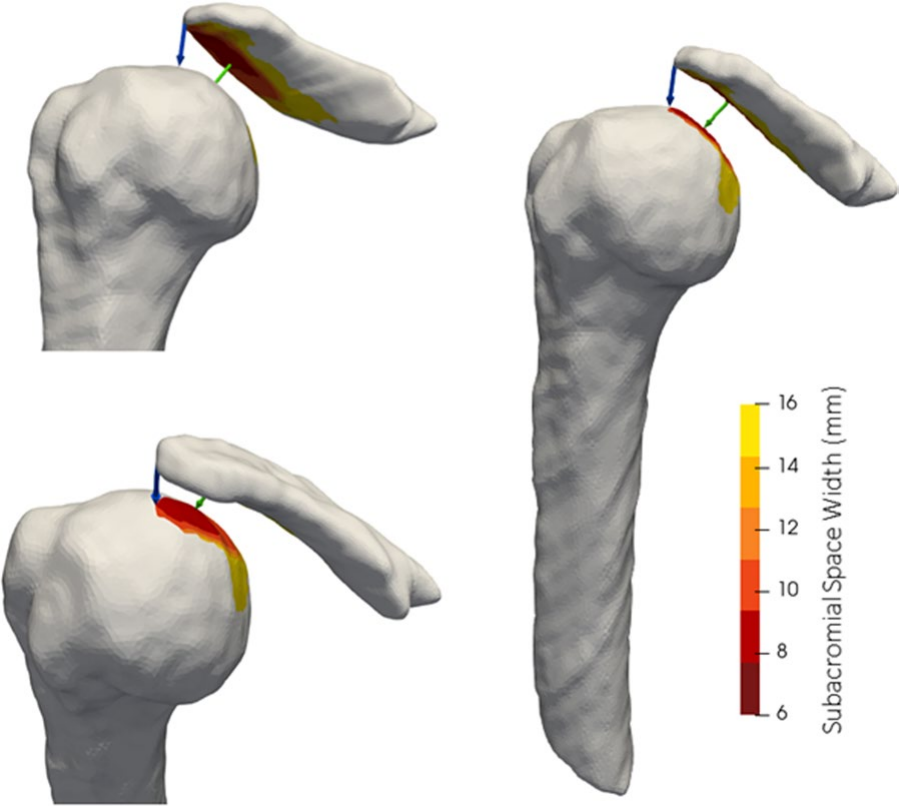
Surface meshes of the humerus and acromion process with heat maps of the distance between the bone surfaces (darker = narrower SAS width; lighter = wider SAS width). The arrows represent the normal projection from the acromion to the closest humeral point (MRI_Hum10; medial-most arrow (green)) and the closest distance between the lateral acromion to the humerus (MRI_Lat; lateral-most arrow (blue))

MRI was acquired by a certified MRI technologist using a 3 T Siemens Tim Trio scanner (Siemens Healthineers, Erlangen, Germany) with a Siemens 18 channel body coil. Participants were lying in a supine position, while coronal images of their right shoulder were captured using a T1 VIBE sequence (FOV = 200 mm, slice thickness = 1 mm, matrix = 256 × 256, voxel size = 0.8 × 0.8x1mm, TR/TE = 11 ms/4.81 ms, flip angle = 10 degrees, average = 1, time = 340 s) (Fig. 1). The humerus and acromion were manually segmented using open-source software (3D Slicer Image Computing Platform, https://www.slicer.org/) [42]. To measure the subacromial space, segmentation post-processing was performed using pyMSKT (https://github.com/gattia/pymskt). Bone surfaces were created by applying a Gaussian filter (*σ*^2^ = 1.56 mm) to binary bone segmentation masks and surfaces extracted using Marching Cubes [43, 44]. Bone surfaces were resampled to 10,000 points using Voronoi clustering [45]. For each vertex on the acromion surface a vector was projected 50 mm normal to the surface, if the vector intersected the humerus the Euclidean distance from the originating point to the intersection was calculated and recorded at the respective vertex. Heatmaps of continuous SAS distances are shown in Fig. 2. Two SAS measures were extracted. First, the mean distance of the 10% closest points (MRI_Hum10) (Fig. 2). Second, the minimum distance from the most lateral point on the acromion to the humerus was calculated as a measure more representative of US-captured SAS width (MRI_Lat) (Fig. 2).

### Ultrasound: training, capture, and outcome measurement

**Fig. 3:**
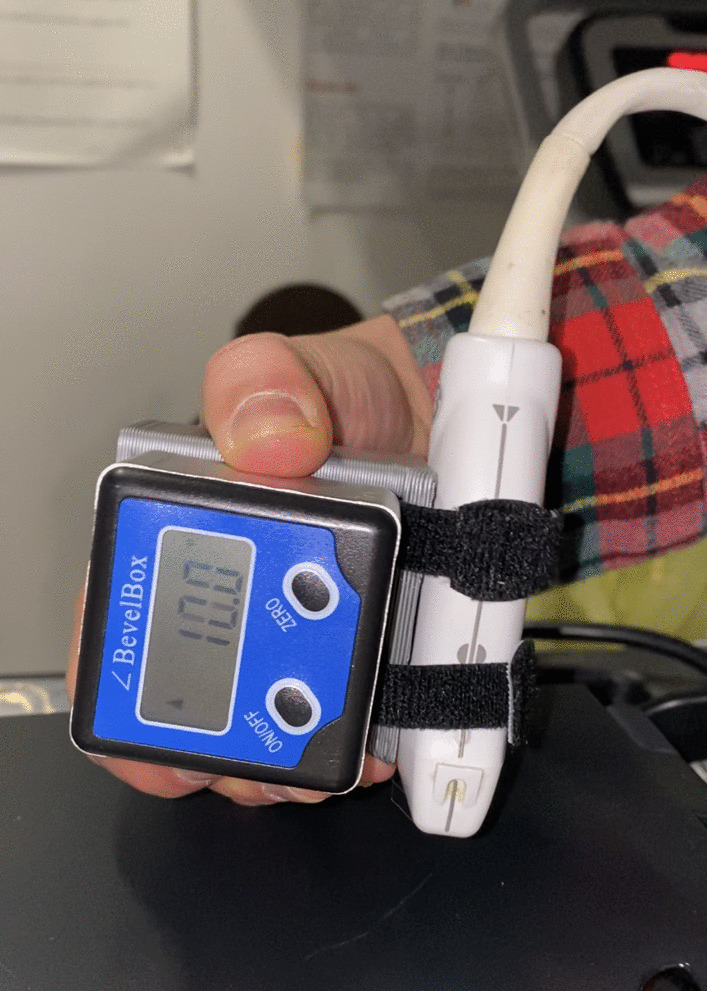
Digital inclinometer housed within a 3D-printed case affixed to the US transducer with Velcro straps

A high frequency (12 MHz) linear array US transducer (GE LOGIQ e, GE HealthCare, Chicago, IL, USA) was used to record US images (Depth, 5 cm; 1 focus point). Images were captured of the right shoulder with the participant in both sitting and supine positions. In the seated position, participants sat with their body erect, arm down by their side in neutral, with their elbow flexed 90°, such that the dorsal aspect of the hand was resting on the ipsilateral thigh [31]. Images were also captured with the participants lying supine with their arm resting in neutral, down by the side with the palm facing medially. Supine images were captured to more closely resemble positioning during MRI acquisition [12]. For each position (seated, supine), the transducer was placed at the most anterolateral aspect of the acromion, with the probe oriented along the long axis of the humerus and perpendicular to the skin [46]. Additionally, a digital inclinometer within a 3D-printed case was affixed to the transducer with Velcro straps [39] (Fig. 3). This permitted capturing the transducer tilt angle of both raters relative to vertical.

US images were captured by one expert rater and one novice rater. The expert rater practiced as a sports medicine physician for 15 years and earned a doctoral degree in musculoskeletal biomechanics specific to the shoulder. The expert also had > 9 years of experience using US to image the shoulder. The novice rater was a graduate student investigator in the field of kinesiology/biomechanics with limited to no experience collecting US images. Prior to participant recruitment, the novice completed introductory US training specific to the shoulder, which included SAS measurements, supraspinatus tendon measurements and supraspinatus and infraspinatus muscle imaging. The training consisted of a written manual and video modules (participant positioning, transducer placement, outcome measurements) prepared by the expert rater and senior author, in conjunction with published research and guidelines. The novice carefully reviewed all written and video modules (~ 4.5 h, which included all tissues), following which 5 h of hands-on practice was completed across four non-consecutive days.

**Fig. 4:**
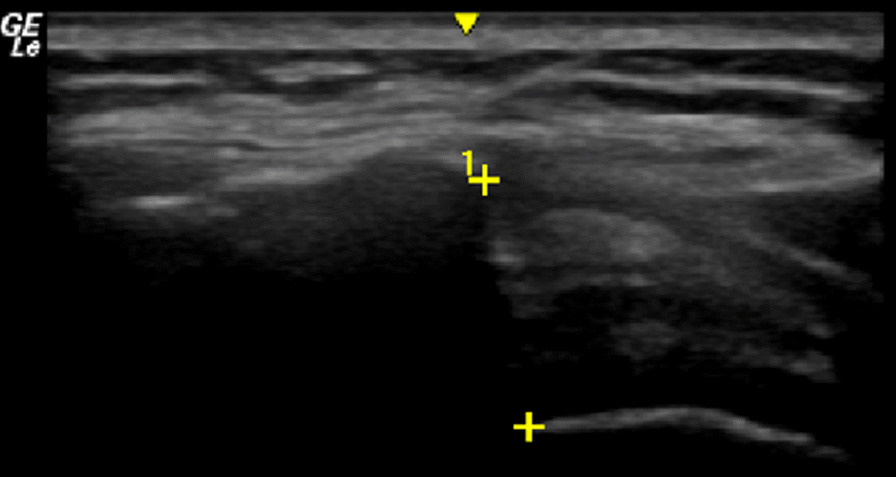
Ultrasound image of the subacromial space. Crosses (+) show the superior (acromion) and inferior (humerus) landmarks of the subacromial space

The raters each performed three repetitions of the SAS measurements with the participant in both seated and supine positions. In addition, the novice rater performed additional SAS measurements at the expert’s transducer angle. All trials were block randomized with raters blinded to each other’s measurements. The novice measurements captured at the expert’s transducer angle were always completed last. The SAS width was measured as the smallest distance between the inferior edge of the acromion and the superior edge of the humerus [46]. All measurements were captured in real time from freeze-framed images (Fig. 4).

### Statistical analysis

Data were analyzed across raters (novice, expert), imaging modalities (US, MRI), body positions (seated, supine), and MRI-based calculation methods (MRI_Hum10, MRI_Lat). The mean of the three repeated US measurements was used for analysis. Intraclass correlation coefficients (ICC) were used to assess absolute agreement of SAS measurements. Specifically, two-way random effects models (ICC (2,1)) assessed absolute agreement between (1) self-selected expert and novice measurements (seated, supine), (2) US and MRI measures (novice, expert; MRI_Hum10, MRI_Lat; seated, supine), and (3) self-selected expert and novice with expert angle (seated). Bland–Altman analyses were also conducted to evaluate agreement between raters and imaging modalities [47]. As well, eight linear regression models ([novice, expert] x [seated, supine] × [MRI_Hum10, MRI_Lat]) were used to determine whether US-based measurements (independent variable) accounted for significant variance in MRI-based measurements (dependent variable). ICCs (2,1) were also used to assess intra-rater reliability of repeated measurements within raters. ICCs were interpreted using classifications previously defined (< 0.50 = poor, 0.50–0.75 = moderate, 0.75–0.90 = good, > 0.90 = excellent) [48]. The magnitude of SAS differences between raters and imaging modalities was additionally calculated. Further, the post hoc power (*p*^2^) for ICCs between novice and expert raters (seated, supine) and between ultrasound and MRI-measured SAS was calculated using a two-tailed test with a null hypothesis of *p* = 0, raters (*k*) = 2, and *α* = 0.05 [49]. All statistical analyses were conducted using StataIC 16.0 (StataCorp LLC, College Station, TX), MATLAB (Mathworks, Natick, MA, USA) and R v4.3.2 [50].

## Results

### SAS: novice versus expert rater

**Fig. 5:**
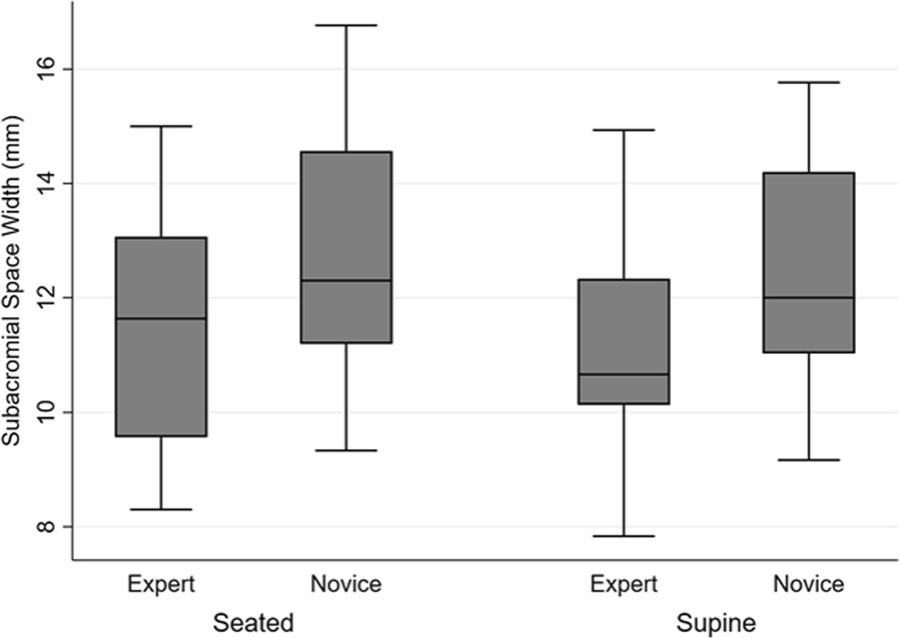
Box plots of the US SAS measures by novice and expert raters for both seated (left) and supine (right) body positions

**Fig. 6:**
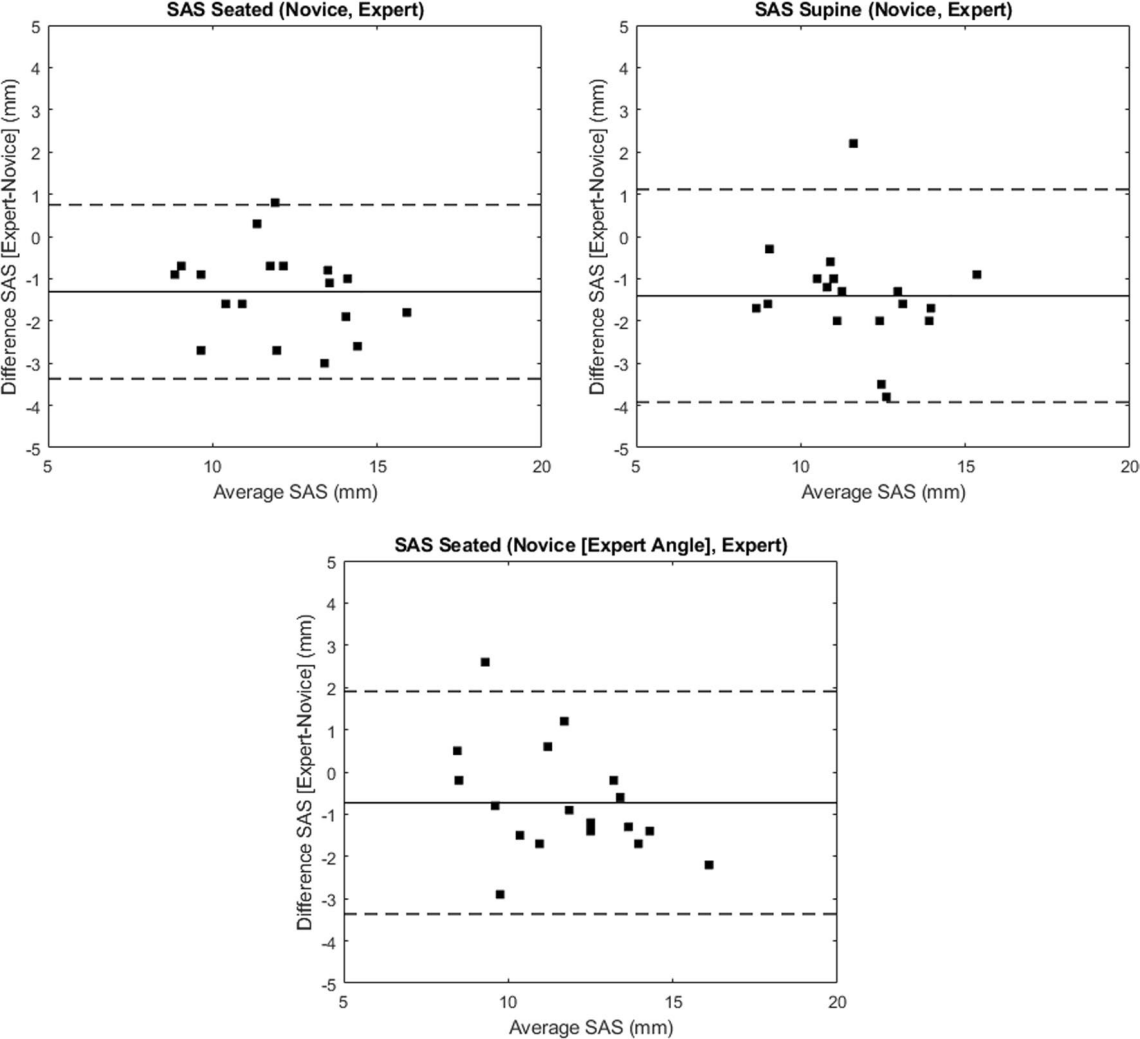
Bland–Altman plots to evaluate agreement between US SAS measurements for novice and expert raters for the seated (top, left) and supine (top, right) body positions, and of the expert (self-selected transducer angle) and novice (with expert transducer angle) in a seated body position (bottom, middle)

The average SAS magnitudes across participants for the novice rater were 12.7 (2.2) mm and 12.4 (2.0) mm for seated and supine positions, respectively, and for the expert rater were 11.4 (2.0) mm and 11.0 (1.8) mm for seated and supine positions, respectively. SAS measurements captured by the novice rater generally overestimated those of the expert rater in both seated and supine positions by 1.3 (1.0) mm and 1.4 (1.3) mm, respectively (Fig. 5). The average absolute differences between the novice and expert were 1.3 (max, 3.0; min, 0.3) and 1.6 (max, 3.8; min, 0.3) for seated and supine, respectively. Bland–Altman analysis confirmed the systematic overestimation of SAS measurements by the novice rater (Fig. 6). ICCs ([95% CI], *p2*) between the novice and expert were moderate, with higher agreement in seated (0.74 [0–0.92], 0.97) compared to supine (0.63 [0–0.88], 0.86).

**Fig. 7:**
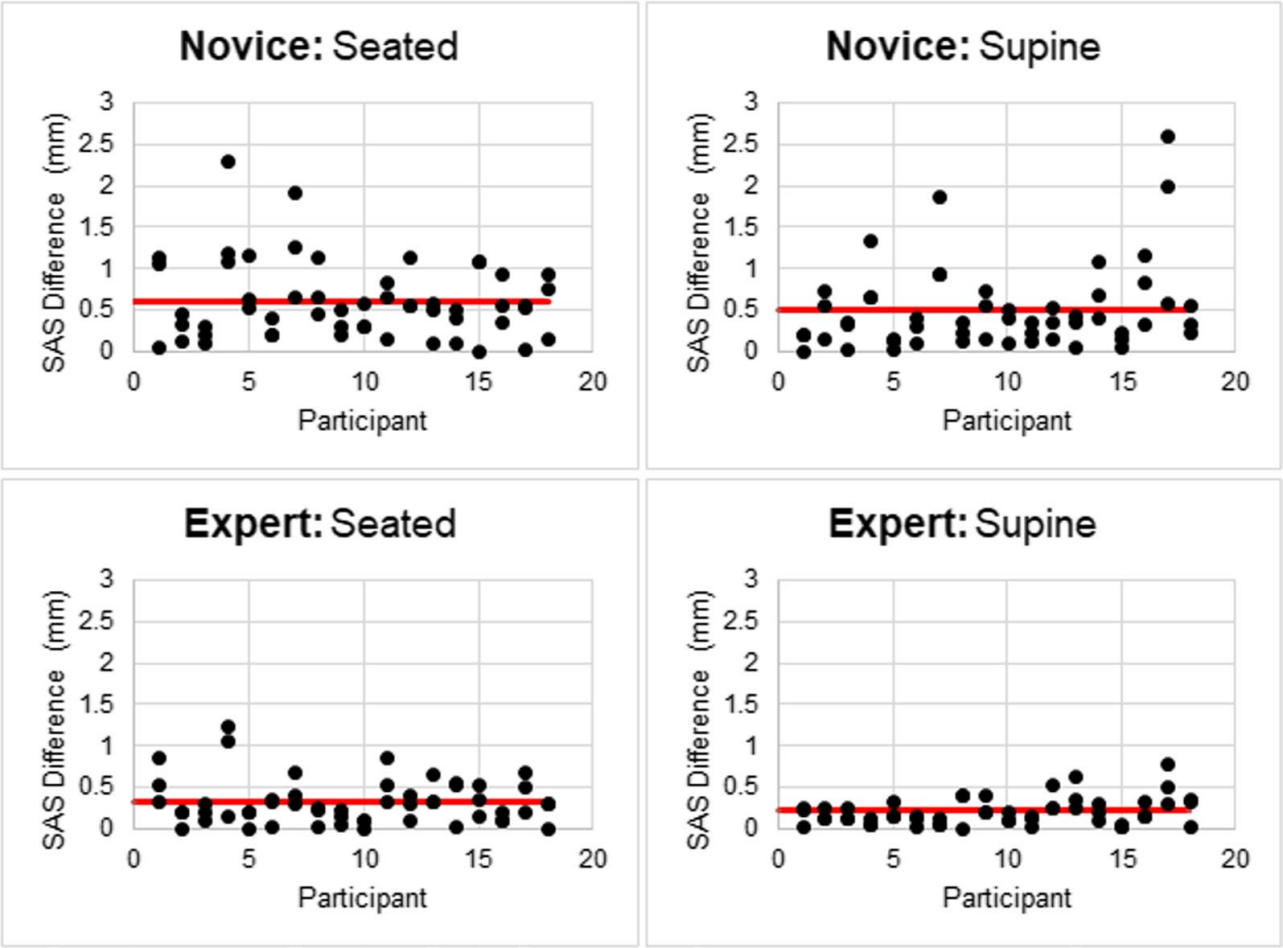
Difference (spread) of repeated ultrasound measurements from the participant-specific mean for each participant for the novice (top) and expert (bottom)

Both novice and expert raters demonstrated good to excellent intra-rater reliability between the three repeated measurements for each condition. ICCs [95% CI] were generally higher for the expert in both seated (0.94 [0.87–0.97]) and supine (0.97 [0.93–0.99]), compared to the novice (seated, 0.83 [0.68–0.93]; supine, 0.84 [0.68–0.93]). The range of measurements about the participant-specific mean for the three repeated measures (absolute value – participant specific mean) was 0–2.3 mm and 0–2.60 mm for the novice rater in seated and supine, respectively, and 0–1.2 mm and 0–0.80 mm for the expert rater in seated and supine, respectively (Fig. 7). Further, both novice and expert raters demonstrated good agreement between their seated and supine measurements, with ICCs of 0.87 [0.68–0.95] and 0.78 [0.51–0.91] for novice and expert, respectively.

### Transducer angle variation

Self-selected transducer tilt angle between the novice and expert raters differed by an average of 9.3 (5.7)° for the seated position and 11.2 (7.1)° for the supine position. Inter-rater agreement was higher when the novice captured measurements using the expert’s mean participant-specific transducer angle (0.79 [0.48–0.92]) compared to their own independent angle (0.74 [0–0.92]) (Fig. 6). The mean difference between raters was 0.7 (1.3) mm.

### Ultrasound and MRI agreement

**Fig. 8:**
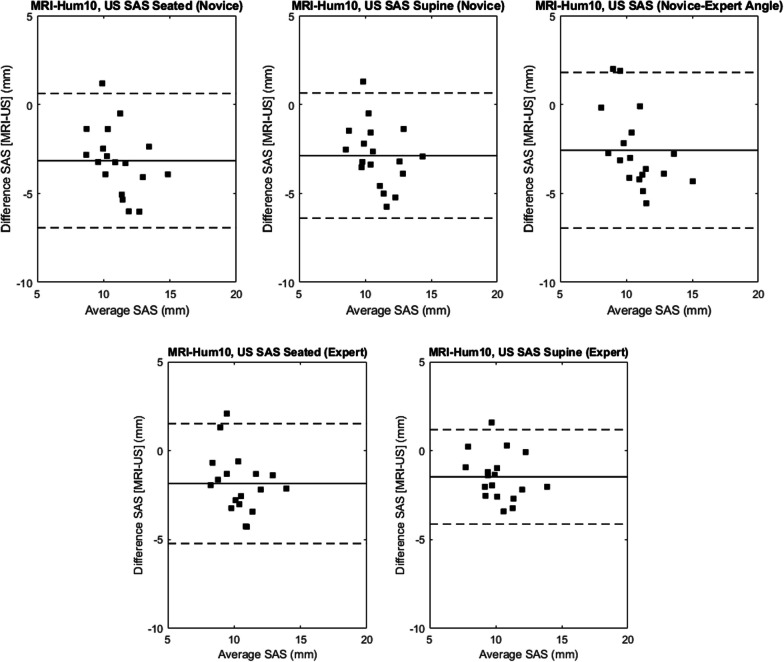
Bland–Altman plots to evaluate agreement between MRI_Hum10 and (1) novice US SAS measurement in seated body position (top, left), (2) novice US SAS measurement in supine body position (top, middle), (3) novice US SAS measurement with expert transducer angle in seated body position (top, right), (4) expert US SAS measurement in seated body position (bottom, left), (5) expert US SAS measurement in supine body position (bottom, right)

**Fig. 9:**
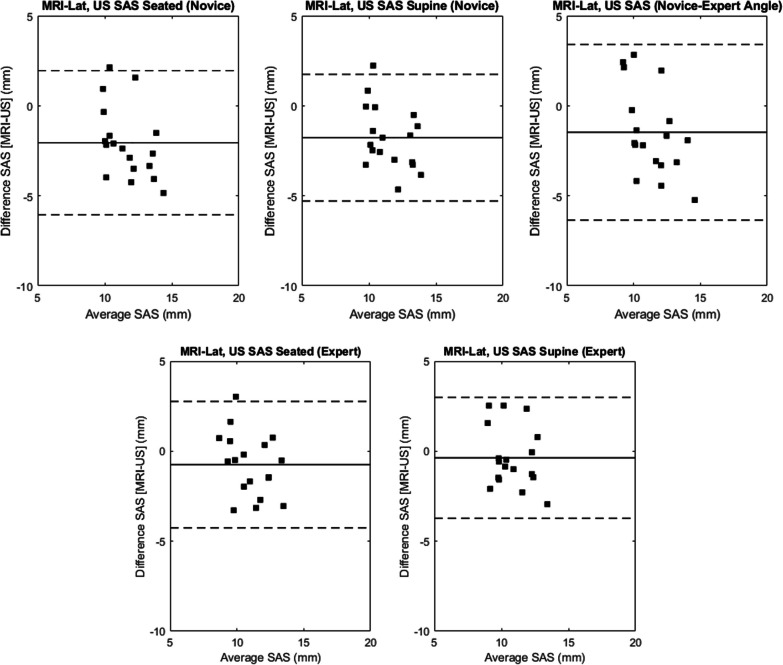
Bland–Altman plots to evaluate agreement between MRI_Lat and (1) novice US SAS measurement in seated body position (top, left), (2) novice US SAS measurement in supine body position (top, middle), (3) novice US SAS measurement with expert transducer angle in seated body position (top, right), (4) expert US SAS measurement in seated body position (bottom, left), (5) expert US SAS measurement in supine body position (bottom, right)

The mean SAS measured from the MRI was 9.5 (1.5) mm for MRI_Hum10, and 10.6 (1.4) mm for MRI_Lat. The agreement between MRI methods was excellent (ICC, 0.98 [0.85–0.99]). Agreement between MRI and US measures was poor for both raters, body positions and MRI-based calculation methods (Table 1). Generally, the US measurements overestimated MRI (Table 1). Bland–Altman analyses revealed a systematic overestimation of MRI SAS magnitudes by US of 1.5–3.2 mm using the MRI_Hum10 method and 0.4–2.1 mm using the MRI_Lat method (Figs. 8, 9).

**Table 1:** Intraclass correlation coefficients [95% confidence interval], post hoc power (*p*^2^) and mean differences (standard deviation) between MRI and Ultrasound

		MRI_Hum10			MRI_Lat			
		ICC [95% CI]	Mean Difference (Standard deviation) (mm)	Power (*p2*)	ICC [95% CI]		Mean Difference (Standard deviation) (mm)	Power (*p2*)
Ultrasound—novice	Seated	0.21 [0.0–0.58]	3.29 (1.62)	0.14	0.27 [0.0–0.62]		2.56 (1.22)	0.21
	Supine	0.23 [0.0–0.60]	3.01 (1.48)	0.16	0.34 [0.0–0.68]		2.11 (1.30)	0.31
	Expert Angle	0.25 [0.0–0.60]	3.02 (1.49)	0.18	0.23 [0.0–0.59]		2.52 (1.25)	0.16
Ultrasound—expert	Seated	0.36 [0.0–0.70]	2.21 (1.09)	0.34	0.45 [0.0–0.75]		1.53 (1.08)	0.52
	Supine	0.49 [0.0–0.80]	1.72 (0.99)	0.60	0.46 [0.1–0.86]		1.46 (0.85)	0.54

Results are presented by raters (expert, novice), body positions (seated, supine) and MRI processing method (MRI_Hum10, MRI_Lat). The novice rater capturing measurements at the expert’s transducer angle is also presented. Mean differences = absolute difference (US–MRI) mm

### Linear regression between ultrasound and MRI

Regression models generally showed US measures of the SAS to be significant linear predictors of MRI (Table 2). The model that accounted for the most variance (*R*^2^ = 0.48) was the expert rater, with a supine body position and an MRI_Hum10 calculation method.

**Table 2:** Regression analysis results (Prob > *F*, *R*^2^) and regression equation for each model (rater, body position, MRI-based calculation method)

		MRI_Hum10 Prob > *F*; *R*^2^; RMSE regression equation	MRI_Lat Prob > *F*; *R*^2^; RMSE regression equation
Novice (ultrasound)	Seated	**p = 0.02; R**^**2**^** = 0.29; RMSE = 1.30** ${\text{MRI}}=4.86+0.37xUS$	*p* = 0.05; *R*^2^ = 0.21; RMSE = 1.30 ${\text{MRI}}=6.83+0.30xUS$
	Supine	**p = 0.02; R**^**2**^** = 0.29; RMSE = 1.30** ${\text{MRI}}=4.63+0.39xUS$	**p = 0.02; R**^**2**^** = 0.28; RMSE = 1.25** ${\text{MRI}}=6.04+0.37xUS$
Expert (ultrasound)	Seated	**p = 0.01; R**^**2**^** = 0.32; RMSE = 1.28** ${\text{MRI}}=4.68+0.43xUS$	**p = 0.03; R**^**2**^** = 0.26; RMSE = 1.27** ${\text{MRI}}=6.48+0.37xUS$
	Supine	**p = 0.002; R**^**2**^** = 0.48; RMSE = 1.12** ${\text{MRI}}=3.23+0.57xUS$	**p = 0.04; R**^**2**^** = 0.23; RMSE = 1.29** ${\text{MRI}}=6.45+0.38xUS$

Significant models (*p* < 0.05) bolded

## Discussion

Moderate agreement was demonstrated between novice and expert raters with the measurements of the novice rater generally overestimating that of the expert. As expected, adopting a consistent transducer tilt angle improved the inter-rater reliability, however, the improvement was small in magnitude. Further, SAS captured using ultrasound overestimated the SAS width measured from MRI. A significant linear relationship was revealed between US and MRI for most measurement conditions, suggesting that US systematically overestimated MRI (0.4–3.2 mm) and prediction models can be generated.

### Novice versus expert SAS measurements

This research evaluated the agreement in SAS measures captured both within and between novice and expert raters using ultrasound. Moderate agreement was demonstrated between raters with higher agreement shown for measurements captured in the standard seated position (ICC = 0.74) compared to supine (ICC = 0.63). The findings of this study are comparable to previous research. Cavaggion et al. [34] found moderate to good inter-rater reliability in both healthy participants (ICC = 0.77) and those with subacromial shoulder pain (ICC = 0.61). The novice rater was a physical therapist with 3 years of research US experience, while the expert was also a physical therapist with 9 years of clinical and US experience [34]. Similarly, Kumar and Attwood [36] found comparable agreement between the novice and expert raters for both left (ICC = 0.70) and right shoulders (ICC = 0.75). The experience of the raters was comparable to the current study, with the novices being physiotherapy students and the expert a registered physiotherapist. An earlier study reported slightly lower inter-rater agreement (ICC = 0.70) between the novice and expert raters when the shoulder position was neutral. The novice rater was an orthopedic resident with limited US experience, while the expert was a musculoskeletal radiologist with 6 years of US experience [37].

While the ICCs between novice and expert raters were classified as modest, the absolute agreement between raters was considerably variable with differences between raters as low as 0.27 mm and as high as 3.77 mm across seated and supine positions (Fig. 6). Other studies have similarly reported variable differences between the novice and expert raters, albeit lower than reported in the current study [34, 36, 37]. Thus, the higher variability demonstrated in this study may be attributed to the relative experience level of the novice rater. While it is possible that variation in transducer positioning may have affected the agreement between raters, the current research found that transducer angle agreement did not have a considerable impact on measurement reliability. While this suggests that transducer tilt variation was not a primary contributor to measurement disparity, the modest improvement in agreement with consistent angles between raters suggests that future research should continue to explore the impact of transducer positioning on measured outcomes.

### Ultrasound versus MRI-based measurements

This study explored the agreement between SAS measured using ultrasound to that captured from MRI. The agreement between imaging modalities was found to be poor (ICCs: 0.21–0.49), with the highest agreement between modalities found between the expert rater (participant lying supine) and the MRI_Hum10 outcome (ICC = 0.49). This finding may have resulted from the better consistency in participant positioning (supine position) during US acquisition [12]. Generally, the US systematically overestimated the MRI SAS measurements for both raters (Figs. 8, 9). Researchers have similarly shown US to overestimate musculoskeletal parameters compared to MRI [51, 52]. For US measurements of the SAS, only the lateral-most borders of the acromion and humerus are visible (Fig. 4). This may not be consistent with the absolute minimum distance between bone surfaces that can be acquired using 3D surfaces reconstructed from MRI. However, interestingly, the agreement between the US measured SAS and the MRI_Lat outcome, which is theoretically more similar to the US measurement, was comparable to MRI_Hum10 located more medially.

### Implications for laboratory-based SAS capture by non-expert

Overall, the current research demonstrated moderate inter-rater reliability, good to excellent intra-rater reliability and poor validity as compared to MRI. The high intra-rater reliability, which is consistent with previous literature [30, 31, 34–37], showed that novice (ICC: 0.83–0.84) and expert (ICC: 0.94–0.97) raters were capable of consistently measuring the SAS in both seated and supine positions, with similar agreement to that reported throughout the literature (ICCs: 0.84–0.99) [33, 35]. However, the poor agreement with MRI suggests that US may not be capable of capturing the true minimum SAS distance, as even a small change in the subacromial space (~ 1 mm) can be clinically meaningful [53]. Despite the poor agreement, the regression and Bland–Altman analyses revealed that a systematic difference between US and MRI exists, with the ability to predict MRI SAS from regression equations (Table 2; Figs. 8, 9). However, it is important to consider that, despite yielding a significant model, the coefficient of determination was low for all models (*R*^2^ = 0.21–0.32), with the exception of the US measure of the expert rater in the supine position, which yielded a stronger relationship with MRI_Hum10 measures (*R*^2^ = 0.48). Thus, with high intra-rater reliability demonstrated, this research suggests that a novice with introductory training may be capable of consistently measuring the SAS width within a testing session. This enables the study of SAS changes as a function of fatigue, exercise, occupational exposures or activities of daily living, sub-maximal and maximal exertions, among others. Further, the true minimum distance, while different in magnitude from that captured using US, may be calculated using regression equations.

## Limitations

Certain limitations of this research should be considered. First, participant positioning during US imaging was standardized across all participants and raters. As participants may have had to change positions (seated, prone) between raters, it is possible that minor differences in posture existed between the two raters, which may have affected the SAS width. As well, with measurements captured from real-time freeze-framed images, it is uncertain whether differences between raters were due to differences in the US image or the measurement protocol. Further, intra-rater reliability of US imaging was assessed from the three repeated measurements in each position. The three measures were captured consecutively as the entire US imaging protocol was blocked randomized. This may have affected the reliability. Lastly, post hoc power calculations for ICC measures revealed a high power between ultrasound measures of novice and expert raters for both seated (*p*^2^ = 0.97) and supine (*p*^2^ = 0.86). However, power was lower for ICCs between ultrasound and MRI (*p*^2^ = 0.14–0.60). Thus, future investigations of image modality agreement should consider a larger sample size.

## Conclusions

Novice and expert raters demonstrated moderate agreement in SAS measurements, which modestly improved with a consistent transducer tilt position. While the agreement between the US and MRI was poor, suggesting that US is not reporting the true minimum SAS, MRI SAS may be predicted from regression equations. Further, high intra-rater reliability suggests that the novice rater with introductory training is capable of capturing SAS measurements. Future research should examine this agreement in older adults with rotator cuff-related pain and/or SAIS where the SAS may be smaller and demonstrate less variability.

## Data Availability

Data available upon reasonable request.

## Abbreviations

CT: Computed tomography
DASH: Disabilities of the Arm, Shoulder, and Hand questionnaire
ICC: Intraclass correlation coefficient
MRI: Magnetic resonance imaging
MRI_Hum10: Mean distance of the 10% closest points
MRI_Lat: Minimum distance from the most lateral point on the acromion to the humerus
SAIS: Subacromial impingement syndrome
SAS: Subacromial space
US: Ultrasound
